# Supplementary material for: Are carbon emissions trading and green financial instruments synergistic? -Comprehensive quantitative research based on content analysis
Source: PLoS One. 2024 Mar 7;19(3):e0298601. doi: 10.1371/journal.pone.0298601 (PMC10919601; doi:10.1371/journal.pone.0298601)
Supplement: S1 Data — (ZIP) [file pone.0298601.s001.zip › Data/地级市Z_F工作报告.zip!/║╙─╧/╨┬╧τ╩╨/└┤╫╘╣1⁄2╓┌║┼í╛┬φ┐╦╩2╛▌═°í┐_2009.docx]

各位代表：

　　现在，我代表市人民政府，向大会作工作报告，请予审议，并请各位政协委员和列席人员提出意见。

　　一、五年工作回顾

　　2004年以来，本届政府在省委、省政府和市委的正确领导下，在市人大、市政协的监督支持下，团结带领全市人民，解放思想，开拓进取，认真贯彻落实科学发展观，积极构建和谐社会，较好地完成了市十届人大一次会议确定的各项任务，开创了经济社会发展的新局面，奠定了新乡跨越发展的坚实基础。

　　2008年，我们认真贯彻落实上级部署，积极应对世界金融危机，努力克服各种困难，保持了经济较快发展的良好态势。全年完成生产总值949.5亿元，比上年（下同）增长13.9%；地方财政一般预算收入48.8亿元、支出99.2亿元，分别增长18.6%和21.2%；城镇以上固定资产投资686.2亿元，增长34%；粮食总产突破74亿斤，连续三年创历史新高；城镇居民人均可支配收入、农民人均纯收入达到13000元和5038元，分别实际增长8.3%和8%。回顾过去的五年，各项工作都取得了新的成绩，新乡发展已迈上了一个新台阶。

　　——过去五年，是整体实力与城市形象大幅提升的五年。五年间，生产总值年均增长14.8%，财政一般预算收入年均增长25.7%，城镇固定资产投资年均增长47.3%，总量分别比上个五年增长了1.6倍、2.1倍和4.8倍；城镇居民人均可支配收入、农民人均纯收入均翻了一番；城镇登记失业率控制在4%以内，人口自然增长率控制在6‰以内。相继获得国家卫生城市、国家园林城市、国家森林城市、中国优秀旅游城市、全国创建文明城市工作先进市、国家知识产权试点城市、全国加工贸易梯度转移重点承接地、中国城市综合竞争力百强城市、中部最佳投资城市、中国金融生态城市等一系列含金量较高的荣誉，连续三届荣获“全国质量兴市先进市”、连续四届荣获“全国双拥模范城”称号。

　　——过去五年，是发展质量明显提高的五年。2008年与2003年相比，税收收入占财政收入的比重按可比口径计算提高了6.1个百分点，达到70.5%;人均GDP、人均财政收入分别增长1.6倍和2.1倍；金融机构存、贷款余额达到759.6亿元和506.2亿元，分别增长73%和69%，贷款余额五年内有四年位居全省第四位；优质专用小麦占麦播总面积的81%，提高12.7个百分点；畜牧业产值占农业总产值的比重提高8.8个百分点。品牌农业实现突破性进展，农业产业化水平明显提高，优质小麦、优质大米、优质中药材畅销全国，无公害农产品和绿色食品认证继续保持全省前列。2008年底，规模以上工业企业和年产值超亿元工业企业分别达到1110家和261家，比2003年增加474家和210家；拥有12家省百户重点企业和6家省50户高成长型企业，数量分居全省第二位和第三位。服务业总量扩大，五年间社会消费品零售总额翻了一番，国内游客接待量、旅游收入年均增长23.1%和25.2%。科技创新能力稳步提升，省级以上企业研发中心达到86家，占全省近十分之一；专利申请量连续五年居全省前三位；2008年高新技术产业增加值占全市工业增加值比重达28%，连续五年居全省前四位；高新技术产品出口占全市出口总额的79％，占全省近30%。五年新增中国名牌产品6个、中国驰名商标3个、国家免检产品13个、省名牌产品44个；中国名牌产品、省级名牌产品数量分别居全省第四位和第二位。

　　——过去五年，是发展方式切实转变的五年。坚持走产业集群发展道路，2008年全市七大产业集群实现销售收入622亿元、利税76.8亿元，占规模以上工业企业的50.2%和60.8%，长垣起重机械产业集群入选 “中国产业集群品牌50强”。产业集聚区建设成效显著，拥有省级以上工业园区5个、省定首批产业集聚区13个，数量分别居全省第一位和第四位。重视科研支撑，积极打造桥北新区河南科技城，省农科院和省科技厅所属科研机构生产试验基地及一批重大科研项目进展顺利。注重集约、节约利用土地，五年来，“三项整治”累计开发土地2.8万亩，连续九年实现占补平衡；已建和在建标准化厂房200万平方米，入驻企业158家。狠抓节能减排，先后关闭了总规模78万吨的15家造纸企业，淘汰了总产能1190万吨的126条水泥生产线，拆除并实施关停改造了55.5万千瓦的45台小火电机组；一批污水处理厂、垃圾处理场、粪便处理场以及医疗废物处理场建成投用；4家单位纳入省级循环经济试点，赵固循环经济产业园正式起步；完成退耕还林49.3万亩，太行山绿化12.4万亩，凤凰山省级森林公园具备接待游客条件，全市林木覆盖率达23.1%；与2005年相比，2008年单位生产总值能耗降低13.4%，化学需氧量、二氧化硫排放量分别下降8.9%和4.6%。

　　——过去五年，是发展后劲显著增强的五年。五年累计完成全社会固定资产投资2312亿元，投资额连续六年居全省第四位；累计实施千万元以上项目3924项，其中亿元以上268项。全市铁路客运量、货运量居全省第二位。规模以上工业增加值、销售收入和利税年均分别增长23.9%、33.2%和42.4%，其中增加值增速连续两年保持全省前三位。企业产品市场影响力不断扩大，金龙集团铜管产量居世界第一，华兰公司血液制品产量居全国第一，华星药业青霉素占国内市场份额的70%，新飞集团散装水泥车、冷藏运输车和新航集团动力转向器、汽车空调、滤清器的产量均居国内前三位，太行振动成为全国规模最大的振动机械企业，卫华集团桥式起重机、电动单梁起重机产量居国内第一，我市基本占领了全国50吨以下起重机及其配件市场。华兰实现上市及再融资，心连心化肥在新加坡成功上市，我市上市企业数量增至3家。证券业、保险业发展迅猛。五年累计新增外商投资企业202家、实际利用外资6.2亿美元，分别比上一个五年增长2.7倍和2.8倍；累计实现进出口总额45.3亿美元，比上一个五年增长4.8倍。美国高盛、英国联合生命、日本丸红、泰国正大、澳门宝龙、上海宝钢、中粮集团等13家世界500强、3家华商500强、21家国内500强企业落户新乡。整合政府资源，组建市投资集团，健全担保体系，投融资平台不断壮大。国企改制取得重大突破，五年累计改制企业150户，占任务数的96%。新增民营企业5.8万家、个体工商户2.6万户，非公有制经济占GDP的比重达到68.9%，五年提高20个百分点，经济活力不断增强。

　　——过去五年，是统筹城乡扎实推进的五年。整体推进新农村建设、县域经济发展、都市区建设“三位一体”系统工程。1367个村达到市级生态文明村标准，首批启动的127个新型农村住宅社区已完成建筑面积250万平方米，入驻农户10292户。沼气入户率39.5%，居全省第一。农田水利基本建设连续四年获得省“红旗渠精神杯”。2008年县域经济总量已占全市的71.2%，比2003年提高2.3个百分点，辉县市、新乡县、长垣县入选中部县域经济竞争力百强县（市）。新增高速公路226公里，通车总里程306公里；4条环路和8条快速路建成通车，30分钟通勤圈初步形成，成为豫北地区唯一的国家公路运输枢纽城市。开展“新城杯”竞赛，推进旧城和城中村改造，县城和重点镇面貌有较大改观。新建、扩建市区道路68条，改造整治小街巷230条，人均绿地面积由2003年的6.5平方米增加到2008年的8.6平方米。市区新增人口21万，城镇化率达到39.2%，五年提高5.7个百分点。

　　——过去五年，是社会事业全面进步的五年。顺利通过国家义务教育“两基”工作验收，改造农村中小学校舍120万平方米，全部消除农村学校D级危房，整合撤并中小学554所，教育资源布局不断优化，进城务工人员子女入学应收尽收；本科层次的新乡学院成功组建，大专层次的新乡职业技术学院已经省政府批准，新乡幼儿师范高等专科学校、医学高等专科学校筹建工作加快推进。市中心医院外科病房楼、市一院综合病房楼、疾病预防控制中心建成投用；全部完成乡镇卫生院改造，新建改造标准化村级卫生室2282所。食品、药品安全工作切实加强，农产品质量安全监测检验体系初步建立。东方文化商业步行街成为城市新的亮点，文化产业投资公司组建成立，市场化运作的电视剧《大长垣》取得成功，潞简王墓申遗工作积极推进，比干诞辰纪念活动影响日益扩大。成功举办两届全市运动会。精神文明建设不断加强，确定了“厚善、崇文、敬业、图强”的城市精神和市徽、市歌，援助四川地震灾区的捐款数额居全省第二位，彰显了新乡企业和人民的博大爱心。信访工作常抓不懈，安全生产形势平稳，社会治安大局稳定，民族团结不断巩固，应急管理能力逐步增强，连续三年荣获“全省平安建设先进市”称号，全国社会治安综合治理优秀市通过验收，人民群众安全感指数位居全省前列。新闻出版、防震减灾、统计、史志、宗教、外事、侨务、档案、气象、人防和民兵预备役建设等工作都取得了新的成绩。

　　——过去五年，是改善民生收效明显的五年。城镇累计新增就业38.6万人，下岗失业人员再就业16.8万人；培训农村富余劳动力105万人，转移就业460.3万人次。在全省率先实行企业基本养老保险、失业保险市级统筹，国有企业下岗职工基本生活费和企业离退休人员基本养老金按时足额发放；加大城乡低保投入，11.9万城市居民和14万农民受益。全面落实农村义务教育阶段中小学“两免一补”政策，免除城市义务教育阶段学杂费。在全国率先实施全市域新型农村合作医疗，参合率达到99.1%。启动城镇居民基本医疗保险试点，已参保42.2万人。累计竣工商品房、经济适用房530.5万平方米和58.6万平方米，廉租住房惠及困难群众1.5万户，市区人均住房建筑面积达到31.8平方米，比2003年增加7.9平方米。开展和谐社区和敬老模范城创建工作，城乡社区建设走在全省前列，新建改造农村敬老院62所。新增农村饮水安全人口74.3万。农村15万人实现脱贫。市区集中供暖达到640万平方米，市区燃气普及率达86%。居民消费层次不断提升，每百户拥有汽车10.2辆、移动电话171.5部，分别比2003年增长14倍和2倍。省市每年向群众承诺的20件实事均按期圆满完成。

　　——过去五年，是政府建设切实加强的五年。落实首问负责制、服务承诺制和限时办结制，精简审批事项290项，市行政服务中心承诺件办理时限平均提速63%，“行政效率提速年”和“两转两提”活动取得良好成效。严格行政效能监察制，开展科室季评活动，完善权力公开运行和监督机制，机关工作规范化管理扎实推进。大力推进行政管理体制改革和机构改革，在全省率先成立公共资源交易中心，建立了乡镇便民服务中心。积极推进乡镇机构改革，合并乡镇29个，精减人员8403名。自觉接受人大法律监督和政协民主监督，累计办理人大、政协议案、提案和建议2598件。强化审计监督，财政资金使用效益不断提高。加强廉政教育，健全工作制度，反腐败力度不断加大；狠刹奢侈浪费，严惩不正之风，倡导真抓实干，政风建设取得积极进展。完善民主决策程序，健全责任、有序、高效的工作推进机制，强化行政问责，各级各部门克难攻坚、狠抓落实的能力进一步提高。

　　各位代表！

　　发展来之不易，成绩属于人民。政府工作的所有成效，都归功于全市人民群众的辛勤付出，得益于历届四大班子奠定的扎实基础，饱含着社会各界的支持帮助。在此，我代表市人民政府，向各位人大代表和政协委员，向工作在全市各条战线上的广大工人、农民、知识分子、干部、驻新解放军指战员、武警官兵、公安干警及社会各界人士致以崇高的敬意！向关心、支持新乡发展的朋友们表示衷心的感谢！

　　反思过去的五年，新乡发展中仍有许多薄弱环节，政府工作中仍有许多不足之处，主要是：经济结构性矛盾比较突出，转变增长方式的任务依然艰巨，创新能力仍需加强，整体实力晋位升级步伐不快；农业基础仍较薄弱，农民持续增收比较困难；当前企业困难较多，财政状况不容乐观，保增长的任务十分艰巨；公共服务依然滞后，社会事业发展与群众需求的差距较大，上学就医、住房改善任务繁重，就业社保压力较大，影响稳定的因素仍然较多；政府工作作风、办事效率仍需转变和提高，反腐倡廉工作有待进一步加强。这些问题，需要我们去认真对待、努力解决。

　　回顾过去的五年，我们有四点深切体会，这既是对以往工作的总结，也必将对今后发展提供有益借鉴。一是必须牢固树立正确的发展理念。在科学发展观的指导下，结合新乡实际，强化以人为本的理念，把人民群众的利益视为最高利益，把人民群众的需要作为一切工作的出发点和落脚点，凡是符合人民群众根本利益的工作，即便困难再大，我们也要全力以赴地组织好、落实好；强化跨越发展的理念，打破常规，拉高标杆，抢抓机遇，冲破束缚，推动新乡跨越式发展；强化改革创新的理念，勇于破除体制障碍，创新发展机制，优化发展环境，增强发展动力；强化资源优化配置的理念，着眼新乡发展大局和长远利益，敢于冲破传统观念和体制的束缚，使政府资源变分散为集中、化零乱于整合，发挥最大效益，进一步解放和发展生产力。二是必须坚持正确的发展思路。思路决定出路。继续推广已经实践证明的好做法，着重抓好对全局具有巨大推动作用的重点工作。紧紧抓住项目建设这个龙头，坚持不懈地实施引资项目双带动战略；突出工业主导地位，加快结构调整，发展产业集群，做大做强工业经济；围绕农业增效、农民增收，抓好“三农”工作；加快服务业发展，努力打造经济发展的双引擎、双支柱；统筹城乡发展，搞好产业集聚区和新型农村住宅社区建设，加快城乡一体化进程；强力推进节能减排，转变经济增长方式，促进可持续发展；高度重视民生改善，促进社会事业发展，确保社会大局稳定。三是必须解决好制约发展的瓶颈问题。对于制约经济社会发展的土地、资金、环境容量、科技创新、产业结构、发展模式、经营机制、重要基础设施等关键环节，按照科学发展观的要求，遵循现代经济规律，高度关注、大胆探索、善于借鉴，不断完善打破瓶颈制约的有效对策，切实解决好这些问题，扫除发展障碍。四是必须积极构建有利于跨越发展的体制机制优势。和其他城市相比，新乡没有自然资源优势，要赶超他人，必须不断彰显体制机制及环境优势，做到人无我有、人有我强。坚持抓好经济运行调节、统筹城乡发展、加强工业经济管理、深化投融资体制改革、完善政府工作推进机制、强化权力运行监督、优化发展环境等工作，营造体制机制独特优势，增强内源动力，为实现跨越发展提供强劲支撑。

　　二、今后五年的工作

　　今后五年，是我市应对复杂局面、推进跨越崛起的关键五年。世界金融危机的深层影响、区域竞争的日益加剧，使我市面临着前所未有的压力和挑战；国家宏观调控措施的不断出台与落实显效，中原崛起的伟大变革与强力带动，为我市带来了难得机遇和巨大希望；发展基础的不断巩固，发展优势的逐步凸显，为我市跨越发展增添了现实动力和强劲信心；整体实力仅居全省中游的现状，人民群众建设美好家园的企盼，赋予了新一届政府更加光荣而又艰巨的历史使命。我们相信，只要全市上下坚定信心、应对得当，完全能够变压力为动力、化挑战为机遇、战胜各种困难，在新一轮发展中抢占先机、赢得主动，使新的五年成为提升发展质量、加快发展速度、实现人民群众美好愿望的五年！

　　今后五年政府工作的总体要求是：以邓小平理论和“三个代表”重要思想为指导，认真实践科学发展观，按照“科学发展、跨越发展、文明发展、和谐发展”的总体取向，加强项目建设，转变发展方式，优化产业结构，增加居民收入，倾力打造中原地区先进制造业基地、高素质人力资源培育基地和区域现代物流中心、现代农业示范中心，建设效益新乡、创新新乡、生态新乡、和谐新乡，力争在统筹城乡、产业集群、产业集聚区、品牌农业、科技创新、生态建设、人力资源培育等方面率先实现突破，综合实力进入全省第二方阵前列。今后五年经济社会发展的主要预期目标是：地区生产总值年均增长11%以上，2013年突破1600亿元；地方财政一般预算收入年均增长11%左右，2013年突破80亿元；全社会固定资产投资年均增长15%，2013年突破1500亿元；城镇居民人均可支配收入、农民人均纯收入年均分别增长8%，2013年分别突破19000元和7400元；人口自然增长率和城镇登记失业率分别控制在6.5‰和4.5%以内；城镇化率达50%以上。为实现五年工作目标，要以统筹城乡发展为主线，在以下四个方面取得突破。

　　坚持科学发展，经济质量要显著提升。调整优化产业结构，提升农业，做强工业，做大服务业，联动推进新型工业化、新型城镇化和农业现代化；强化“三农”工作，狠抓品牌农业，把我市建设成为在全国有重要影响的农产品生产基地、加工基地和种子基地；切实转变发展方式，加强科技创新，实施名牌战略，加快企业重组，壮大产业集群，增强产业集聚区集聚效应，提高产业集中度；培育战略支撑产业，加快“一谷五基地”建设，改造提升传统优势产业，促进产业升级，提高区域竞争力，建设效益新乡。不断优化产业结构，销售产值突破百亿元的产业集群、产业集聚区和大型企业集团力争达到4个、7个和8个。六大战略支撑产业主营业务收入、高新技术产业增加值分别占全市规模以上工业增加值的70%和33%以上，工业利税年均增长20%以上，税收收入占财政一般预算收入的比重力争超过全省平均水平。实施知识产权战略，争创一批中国驰名商标和中国名牌产品。

　　推进跨越发展，综合实力要明显增强。改革体制机制，破解发展瓶颈，促进经济总量跨越增长，力求做到好中求快、更好更快。创新统筹城乡发展机制，努力在建立城乡一体的土地、户籍、社保、基础设施、金融等体系方面取得突破，基本建成329个新型农村住宅社区；创新项目建设推进机制，在招商引资的力度与广度、项目建设的数量与质量上取得突破；创新经营组织方式，在建立现代企业制度、促进土地流转、发展农民专业合作组织和现代服务业等方面取得突破；创新融资机制，在密切银企合作、壮大投融资平台、引进风险和产业投资、发展地方金融机构、促进企业上市、健全担保体系等方面取得突破；强化自主创新，优化发展环境，在提高企业核心竞争力、提升区域环境软实力等方面取得突破，力争建成全国科技进步先进市和国家级高新技术产业开发区，建设创新新乡。五年中，固定资产投资总量保持全省前四位，力争引进亿元以上的重大项目110个以上、三个“500强”企业20家以上，新增上市企业10家左右。

　　强化文明发展，生态环境要根本改善。狠抓节能减排与生态保护，抓好专项整治，提高环境容量，增强可持续发展能力，提升城乡生态文明整体水平。加强林业生态建设，继续推进生态文明村、生态防护林、生态廊道网络建设；开展节能减排全民行动，发展循环经济，推动清洁生产；从根本上解决市区水源匮乏和全市地下水位日益下降问题，做好漏斗区补源工作，加快新乡市区水系景观建设；加强城乡环境综合治理，完善城市管理长效机制和农村生活垃圾日常管理机制，加大环保基础设施建设，促进生态系统良性循环，争取建成国家环保模范城，建设生态新乡。河流出境断面水质基本稳定达标，空气质量良好级以上天数达到85%以上；林木覆盖率达到29%以上，90%的村庄达到生态文明村建设标准，沼气入户率达到50%以上，单位生产总值能耗明显降低，生态环境质量居全省前列。

　　促进和谐发展，惠民措施要更加务实。坚持以人为本、富民为先，实现民生改善和经济发展双赢，争取建成全国文明城市和国家健康城市，建设和谐新乡。整合政府可控资源，加大投入力度，强化城乡公共基础设施的共建共享，加快城市危旧住宅区和城中村改造，推进新区教育、医疗、体育、商贸等公共服务设施建设，改善人民群众生产生活条件。加快新型农村住宅社区建设，在有条件的地方率先实现农村居住环境、公共服务、就业结构、消费方式城市化；大力发展社会事业，促进社保体系更加健全、城乡教育均衡发展、医疗卫生协调发展、城乡文化日益繁荣、科学普及深入广泛、体育健身蓬勃兴盛；完善群众工作长效机制，深入开展平安建设，确保社会大局稳定。

　　三、2009年的工作

　　2009年将是本世纪以来全市经济发展最为困难、财力最为紧张的一年，也是蕴含重大机遇的一年。今年的政府工作，必须把保增长放在首位，坚定信心，振奋精神，正视困难，抢抓机遇，全力抓好落实，奋力晋位争先，打好保增长、保民生、保稳定、调结构的攻坚战，确保经济平稳较快增长，确保跨越发展的良好态势。全年主要预期目标是：地区生产总值增长11%；全社会固定资产投资增长21.5%；地方财政一般预算收入增长11%；城镇居民人均可支配收入、农民人均纯收入分别增长8%；人口自然增长率、城镇登记失业率分别控制在6.5‰和4.5%以内；单位生产总值能耗降低4.6%，化学需氧量、二氧化硫排放量分别下降8.9%和8.1%。完成全年目标，必须着力抓好九个方面的工作。

　　（一）强化经济运行调节，全力以赴为企业排忧解难。高度重视企业的市场经济主体地位，密切关注经济形势变化，加强对重点行业、骨干企业和主要产品的监测分析，及时解决倾向性、苗头性问题，带动全市经济健康快速增长。建立政府、银行、企业间贷款风险评估机制，完善救助措施，防止因简单收贷导致企业资金链断裂。年内召开银企洽谈会不少于4次，力争新增贷款70亿元。支持大型企业发行企业债券、公司债券和中期票据，鼓励中小企业发行集合债券。建立我市产业投资基金。应用好期货等现代金融工具，促进经济发展，降低经营风险。大力发展典当、担保行业，引导社会资本投资设立担保机构，扩大小额贷款范围。规范壮大地方金融机构，增加邮政储蓄银行贷款数量。加快投资集团发展，强化投融资能力。挖掘土地潜力，集约、节约利用土地，确保重点项目建设用地。围绕企业需要，扎实开展“企业服务年”活动，坚持特事特办、特时特办，提高办事效率，提升服务水平，优化发展环境，千方百计帮助企业解决各种难题，力保困难骨干企业资金链不断、品牌不倒、主要客户不丢、技术骨干不流失，面上企业重点项目建设、自主创新、产品开发、产品结构调整、企业重组、企业上市工作不停顿。

　　（二）扩大投资和消费需求，保持经济发展良好态势。牢牢抓住国家扩大内需的政策机遇，加快项目谋划与建设，增强投资和消费对经济增长的拉动作用。加大项目争取和引资力度。围绕三个“500强”投资趋向，有效承接先进制造业和现代服务业梯度转移，积极开展产业链招商、以商招商、园区招商和重点项目定向招商，力争在重大工业项目、科研基地、文化旅游、商贸、物流、教育、房地产等方面取得新突破。依托庆祝建市六十周年活动，承办好各类经贸洽谈，积极参加各项招商活动，全年力争新批外商投资企业20家，实际利用外资2.4亿美元、市外资金150亿元以上。加快重大项目建设。计划实施千万元以上项目974个，总投资1632亿元，年度完成投资520亿元。实施重点项目162个，总投资796.1亿元，年度完成投资203.8亿元。开工胡韦公路与黄河公铁两用桥连接线、济东高速黄河大桥、东高庄互通立交新建工程等干线公路建设项目。开工建设渠东2×30万千瓦热电联产、赵固2×30万千瓦综合利用电厂、金龙新型锂离子电池及锂动车辆、雨润集团200万头生猪加工厂、卫华集团大型港口起重机械项目二期、新亚纸业20万吨轻量涂布纸技术改造等项目。加大安新高速扩建、石武铁路客运专线、新晋高速、南水北调新乡段、桥北新区等基础设施建设项目及孟电集团2×30万千瓦热电联产、新飞2万辆专用汽车和汽车电子、中新化工40万吨甲醇等重大工业续建项目的投资力度。力争心连心化肥40万吨尿素、赵固一矿240万吨原煤、新航集团20万台电动转向器等一批重大项目竣工投产。谋划引进一批高质量项目。突出抓好127个重大项目的谋划工作，不断提升项目投资规模、科技含量及产业带动关联度，严控高污染、高耗能项目上马。加快新中益2×100万千瓦火电机组、宝山二期、宝泉二期、金龙铝代铜、华亚铸钢2×1千方高炉等重大项目的前期工作，做好煤化工基地的规划、争取工作。切实加快服务业发展。促进新乡粮食物流园区、小店物流港、新钢物流园等现代物流园区建设，争取深圳华强和嘉年华动漫设计与展示园、美国双蓝公司医疗保险试点、海宁皮革城连锁市场、家乐福购物中心、红星美凯龙家居广场等项目落户新乡。加快发展保险、证券、中介服务业，支持农村供销社加快组织创新与经营创新，重视新农村现代流通网络工程建设。推进“早餐工程”、便民超市等社区服务业。加快百泉景区改造，争取开工建设南太行旅游环路，将纪念比干诞辰活动办成全省知名品牌。积极扩大居民消费需求。发展消费信贷，落实居民购买汽车、住房等大宗消费品的鼓励政策，继续抓好 “万村千乡”市场工程和“家电下乡”工作，鼓励文化、体育、网络、休闲等热点消费。

　　（三）以增加农民收入为核心，抓好“三农”工作。认真贯彻党的十七届三中全会精神，落实好中央一号文件，深化农村综合改革，抓好农业生产，发展品牌农业，深入推进新型农村住宅社区建设，促进农业增效、农民增收，不断提高农民群众生活水平。高度重视粮食生产。不折不扣地落实好各项支农惠农政策，严格执行小麦最低收购价，调动农民种粮积极性。继续抓好小麦高产开发工程，优质麦种植面积稳定在400万亩以上，同时抓好玉米、水稻生产，粮食总产达到75亿斤以上，建设河南粮食核心区优质高产示范区。提升畜牧业发展水平。大力发展畜禽规模化养殖，深入实施绿色畜牧工程，新认证无公害畜产品10个，奶牛存栏8万头，生猪出栏300万头，肉牛出栏25万头，畜牧业占农业总产值的比重较上年提升1个百分点。加快农业产业化进程。按照农民意愿，推进土地规范有序流转和农业集约经营，探索建立土地收益分配新机制。密切与国内知名龙头企业合作，支持五得利、新良、亚特兰、宏力、克明面业等企业发展，新培育认定市级重点龙头企业20家，引导支持各类农民专业合作组织规范发展。大力发展品牌农业和订单农业，提升农业标准化水平。抓好现代农业示范区建设，加快发展蔬菜、油料、花卉、中药材等高效农业。新增市级以上名牌农产品20个，新增无公害农产品和绿色食品基地认定面积20万亩、认证产品20个，扩大金粒小麦、太空米、迪一米、红提葡萄等名牌农产品的市场占有率。不断改善农业生产条件。抓好农业基础设施建设，加快修复完善引黄灌溉水利设施，规划建设沿黄蓄水工程，扩大引黄灌溉面积，有效补给地下水源。改善井灌、水库灌溉条件，新增节水灌溉面积10万亩。继续实施农业综合开发，改造中低产田11万亩。解决农村17万人饮水安全问题。坚持科技兴农，加快中国农科院新乡县农业生产科技成果转换试验繁育基地建设，建成38个区域农业技术服务中心，不断提高现代农业科技支撑水平。建立市县两级化肥储备制度，提高农机装备水平，推广土壤深耕作业，抓好农业生产。多渠道促进返乡农民工就业。高度重视，强化培训，引导创业促就业，让农民工求职有信息、就业有门路、创业有援助，力争安置返乡农民工及农村新增富余劳动力就业13万人。发挥长垣厨师、长垣防腐、新乡海员、原阳电焊等劳务品牌效应，提高农民转移就业的组织化程度。

　　（四）优化工业经济结构，充分发挥工业的主导作用。应对当前危机，必须加大结构调整力度，构建现代产业体系，壮大产业龙头，延伸产业链条，拓展配套能力，推动工业集聚、集群、集约发展。大力培育战略支撑产业。将冷谷、生物与新医药基地、电池及新型电池材料基地、特色装备制造基地、煤化工基地、汽车及零部件基地等“一谷五基地”作为我市战略支撑产业，集中政府优势资源强力扶持，六大产业全年实现销售收入、利税较上年分别增长25%和20%。按照新型工业化的要求，落实国务院产业振兴规划，改造提升纺织、食品、建材、造纸、能源电力等传统优势产业。大力发展产业集群。发挥行业协会的作用，引导企业加强分工协作，重视公共技术服务平台建设，逐步实现龙头企业以研发、总装、销售为主，中小企业以协作配套为主，提高产业整体竞争力。积极引进国内外战略投资者和行业龙头企业，引导本市企业加快战略重组，优化资源配置，提高产业集中度，增强区域产业竞争力。引导生产要素向优势企业、优势产业、优势区域集中，七大产业集群的发展质量力争显著提升。支持龙头骨干企业加快发展。加大扶持力度，推动80户优势骨干企业做大做强。新增销售收入超亿元企业20家以上，力争金龙集团达到200亿元、新飞集团达到70亿元。强化与中航集团的深度合作，支持新航集团做大做强。太行、新亚、金龙、新飞等企业争取实现上市。扶持中小企业发展。健全中小企业金融担保体系，鼓励金融机构创新中小企业信贷产品，引导中小企业科学定位、调整结构和完善管理，支持其开拓市场、加快发展，增强吸纳就业的能力。

　　（五）强化科技创新，深化改革开放。以科技创新来助推发展方式的转变和区域竞争力的提升，以改革开放来强化抵御风险、跨越发展的能力。不断提高自主创新水平。尊重知识，关爱人才，加大创新投入，实现科研创新与经济发展的有效对接。争取重要的研发基地和高科技项目落地桥北河南科技城，尽快形成集聚态势，发挥辐射效应。突出企业的自主创新主体地位，80户优势企业年内都要组建研发中心，新增省级企业技术中心3家。抓住我市被列为全省唯一“两化”融合试点城市的机遇，促进信息化与工业化深度融合，鼓励企业应用信息技术提升竞争力。发展高新技术产业，用先进适用技术改造提升传统优势产业，申报国家火炬计划新乡生物医药特色产业基地、省高新技术新乡振动机械特色产业基地。进一步推进产学研结合。建立产学研各方优势互补、利益共享的技术合作机制，鼓励企业与科研机构、高等院校共建研发中心，加快科研成果的产业化进程，合作培养创新人才。推动对外科技交流与合作，建立面向全国的技术难题招标工作机制，解决制约我市经济发展的关键技术。扎实推进标准化战略和知识产权战略。建设好国家电池产品质检中心。创建国家级农业标准化示范区1个、省级示范县1个。争创中国驰名商标2个、中国名牌产品2个、省著名商标15个、省名牌产品10个。专利申请保持在1000件以上，发明专利比例达到20%，推动新乡制造向新乡创造转变。加大改革攻坚力度。年内完成市、县两级新一轮政府机构改革。全部完成市属企业改制，积极推进局 （委）属企业改制。加大农村信用社改革，加快市商业银行上市步伐。做好经营性文化事业单位转企改制，完成公益性文化事业单位劳动人事、分配和社会保障制度改革。深化农村改革，加快农贸市场改造升级，探索推进政府和乡村管理的农贸市场改制工作，凡有盈利的市场要逐步民营化。提高对外开放水平。落实扶持企业出口的相关政策，鼓励有实力的企业开展跨国经营。优化进出口结构，多引进高端设备、先进技术，提高机电产品、高新技术产品和优质名牌农产品的出口比重。进出口总额完成13.8亿美元，其中出口8.8亿美元。积极争取设立新乡海关和出入境检验检疫机构。

　　（六）统筹城乡发展，加快推进城镇化。坚持实施“三位一体”系统工程，完善土地利用、城乡和产业集聚区三大规划，健全统筹城乡发展的政策支撑体系，逐步缩小城乡差距。突出中心城市带动。实施市区、桥北新区“双核”城市发展战略，推进新区、开发区、凤泉区、新乡工业园区联体发展，促进主城区东部隆起，加大城市西区改造开发力度，增强城市整体辐射带动作用。开工建设新区外国语中学和市民中心。平原博物院和青少年活动中心 “三合一”工程建成投用，市档案馆完成搬迁工作。运用市场机制，建设迎宾大厦、新闻大厦、商会大厦、新区五星级宾馆等项目。加快火车站广场等十大特色建筑群建设。加大第一批重点推进的21个城中村和10个危旧住宅区拆迁改造力度，确保年内有实质性进展。促进房地产市场发展，市区新开工100万平方米、竣工100万平方米，完成投资30亿元。开工建设学院路北段、宏力大道东段等9条市政道路，打通断头路，完善路网体系。抓好小街巷整治，建设平原路快速公交系统，加强“数字城管”建设，完善城管长效机制。推进城市绿化、美化、亮化工作，启动牧野湖二期及卫源湖建设，做好东湖、贾太湖、凤泉湖前期工作，建设生态宜居城市。深入开展“新城杯”竞赛，使县城和重点镇面貌明显改善。城镇化率达到40.7%。发展壮大县域经济。加快县城及中心镇基础设施建设，改扩建乡村道路650公里。健全县域经济发展的激励奖惩机制，辉县市、新乡县要争取进入全省新型工业化转型示范县，将长垣县作为全市示范县予以支持。培育县域特色产业，大力发展高效农业，壮大民营经济，构筑城乡协调发展的县域经济新格局。加强产业集聚区建设。把产业集聚区作为实现集约发展、加速工业化和城镇化进程的重要载体。科学定位功能，完善服务设施，引导企业和新上项目入驻集聚区。突出抓好省定13个集聚区基础设施建设，年内各级产业集聚区完成基础设施投资20亿元，新上千万元以上项目400个以上。按照省里部署，高起点、高标准规划建设桥北新区，提高胡韦线两侧工业及城镇聚集度，促进新乡与郑州的产业对接和城镇连接。加快新型农村住宅社区建设。落实“以奖代补”政策，支持房屋产权抵押贷款，允许产权在乡镇区域内流动，鼓励农民群众在新型农村住宅社区建房。积极争取上级投资，引导金融部门开展农户建房、购房按揭贷款，破解建设资金投入难题。结合市场化运作，加大学校、卫生室、文化大院等基础设施的投入力度，建设社区服务中心，使城市文明向农村延伸，吸引群众入住。加大首批127个新型农村住宅社区建设力度，今年再启动202个，争取完成建筑面积450万平方米。坚持把异地搬迁式扶贫、南水北调1.6万移民搬迁，与规划的新型农村住宅社区建设统筹安排、统一实施。

　　(七)强化节能减排，建设生态新乡。把污染治理与节能减排作为转变发展方式的突破口，强化环境执法，严格执行节能减排一票否决制，促进可持续发展。高度重视污染治理。加强各县 （市）污水处理厂和垃圾处理场管理，确保正常运营。推进乡镇、中心村污水处理、垃圾处理设施建设，抓好产业集聚区和农村面源污染治理，完善农村生活垃圾日常管理机制，治理规模化畜禽养殖污染，新增农村沼气用户4.5万户。对卫河流域进行深度治理，抓好海河流域和南水北调工程沿线污染治理，建设新乡县二次污水处理厂。深入推进节能降耗。依托与新奥集团等企业签订的节能及新能源合作协议，开展节能技术改造，加快淘汰落后生产设备与工艺。突出抓好年耗能5万吨标准煤以上企业的节能工作，重点支持一批余热余压利用、能量系统优化、工业锅炉及电机系统改造等项目，努力解决我市高耗能行业存在的共性问题，实现关键技术的突破。强化支持引导，积极做好锂电、太阳能、风能等新能源、可再生能源的推广与利用。严格土地管理，积极利用山地、荒地和劣质地建设项目，完成 “三项整治”7500亩，竣工标准化厂房30万平方米。强力抓好生态建设。75%以上的行政村年内要达到生态文明村建设标准。开展大绿化活动，深化林权制度改革，完成工程造林28.8万亩，村镇绿化率达到35%以上，林木覆盖率提高1.8个百分点。抓好济东高速、大广高速、京珠高速、大外环、黄河故道森林公园等生态防护林带建设。加快北部山区采石企业结构调整和资源整合，继续抓好凤凰山省级森林公园核心区绿化美化。建设垃圾发电、沼气发电项目，推进赵固工业园等15个循环经济项目建设，争取更多项目列入省循环经济试点。

　　(八)注重社会建设，着力改善民生。从影响群众切身利益的问题入手，深入开展和谐社会建设，努力提高城乡群众生活质量，全面完成省市承诺的20件实事。进一步扩大和促进就业。就业是改善民生的重中之重，是最基础的工作。必须花大力气认真抓好就业岗位开发和城乡就业培训，搞好企业用工衔接，鼓励企业吸纳更多人员，引导困难企业采取在岗培训、轮岗工作等办法，尽可能不裁员或少裁员。抓好下岗失业人员再就业，加强大中专毕业生、退役军人就业指导，解决好被征地农民及返乡农民工就业问题，鼓励自主创业，建设创业型城市。力争城镇新增就业7万人，下岗失业人员再就业2.4万人。强化城乡社会保障。加大社保扩面征缴力度，增强保障能力。落实国家政策，提高企业退休人员养老金水平。健全以医疗保险、最低生活保障和社会救助为主的农村社会保障制度。积极应对人口老龄化，发展社会养老服务事业。加强敬老院建设与管理，完善服务措施，提高农村五保户集中供养率。加快建设保障性安居工程，新开工经济适用房25万平方米，竣工15万平方米。新建、收购廉租住房2万平方米，扩大城市困难家庭住房保障覆盖面。弘扬社会爱心，发展慈善事业，提高社会救济救助水平。加强劳动监察，维护劳动者特别是农民工的合法权益。加大扶贫攻坚力度，稳步减少贫困人口。促进城乡教育均衡发展。继续推进中小学布局调整，坚持义务教育阶段公办学校免试就近入学制度，使更多家庭享受到优质教育资源。落实农村义务教育经费保障机制，强化基层师资力量，完成10万平方米农村中小学危房改造任务，提高农村教育水平。保障进城务工人员子女、贫困家庭儿童、留守儿童等群体的就学权利。基本普及高中阶段教育。加快职业教育资源整合，进一步做好为驻新高校服务工作，推进本科院校二级学院园区和职业教育园区建设。健全城乡卫生服务体系。完善以市、县 （市）综合医院为龙头、乡镇卫生院为枢纽、中心村（社区）卫生室为基础的城乡公共卫生服务网络，提高社区医疗卫生水平，建成836所标准化村卫生室，不断改善城乡医疗基础条件。在全市二级以上公立医院全面实施单病种限价、设立惠民病房、开展按病种付费等措施，努力解决群众看病难、看病贵问题。鼓励城市卫生医疗机构拓展农村市场，搭建双向转诊平台，开展城乡医务人员互派交流，培养基层卫生人才。扩大和提高新型农村合作医疗的报销范围和报销比例，逐步实现市县乡各级医疗服务机构网络直报，方便参合农民就医。稳步推进城镇居民医疗保险试点工作，参保人数达50万人以上。加大医疗服务机构监管，提高服务管理水平。发展城乡文化事业。推进公共文化服务体系建设，加快中原图书文化产品大世界、潞简王墓申遗、印刷包装产业园等重点文化项目建设，打造大型音乐舞蹈史诗剧《牧野雄风》、力争获得省“五个一”工程奖。加强农村文化大院和乡镇文化站建设，发展广场文化、节庆文化、民间文化、社区文化，丰富群众文化生活。争取成功申报第七批全国重点文物保护单位3处以上。加快体育基础设施建设，开展全民健身运动。做好新闻出版、广播电视、社会科学等工作，促进协调发展。

　　（九）强化公共管理，维护社会稳定。完善重大疾病防控体系，强化公共突发事件应急管理，提高应急处理能力。提升城乡社区服务功能，创新管理体制，创建和谐社区。加强民族团结工作，依法管理宗教事务。支持工会、共青团、妇联等人民团体积极参与社会管理和公共服务。稳定低生育水平，提高人口质量，推进基层计划生育服务设施建设，加强流动人口计生服务管理和出生人口性别比综合治理。抓好防震减灾、气象、人防及审计、统计、史志、外事、侨务等工作。启动法律援助“应援尽援”工作。依法保护妇女、未成年人、残疾人和老龄人的合法权益。落实好老干部的“两个待遇”。强化全民国防观念，支持国防建设，提高民兵预备役和武警、消防部队建设水平，争创全国双拥模范城“五连冠”。发挥基层民事调解组织作用，建立信访稳定长效机制，力争信访总量退出全省前五位。强化安全生产隐患排查监管，坚决遏制重特大事故发生。抓好食品药品和餐饮卫生的日常监管，打击违法经营活动，确保群众健康安全，建设“食品安全市”。加强社会治安综合治理，保持“严打”高压态势，强化治安防范，打造平安新乡。

　　四、加强政府自身建设

　　各级政府必须增强使命意识，切实履行职责，努力建设人民满意的服务型政府、效能政府、法治政府、务实政府和廉洁政府。

　　坚持民本为上。政务就是服务。要增强宗旨意识、责任意识和服务意识，把人民群众的需求作为改进政府工作的第一信号，带着对人民群众的深厚感情去思考发展、决策工作、处理问题。高度关注群众的安危冷暖，围绕群众、企业和基层的需要来开展工作，努力解决土地征用、房屋拆迁、企业改制等过程中群众反映的突出问题，切实增加居民收入，改善生产生活环境，提高人民群众的生活质量。当前形势下，更要特别关注和解决好就业、困难群体的生活等民生问题。

　　坚持效能为先。效率高低直接体现着政府的执行能力。要将决策咨询、专家论证和公众参与相结合，切实提高科学民主决策水平。转变政府职能，全面推进政企、政资、政事分开，切实解决政府与市场、社会之间的越位、错位和缺位问题。完善政府运行机制，精简审批事项，落实全程代理制、绩效考核制，提升公务员素质，加强效能监察，强化行政问责，促进政务提速，逐步形成市场配置资源、社会自主管理、政府科学调控的善治格局。

　　坚持法治为纲。依法行政是政务工作的基本要求。要落实行政执法责任制，畅通行政复议渠道，提高执法质量，努力做到有权必有责、用权受监督、侵权要赔偿、违法要追究，促进社会公平正义。加强政务公开，自觉接受人大的法律监督和政协的民主监督，认真负责地办理人大、政协的议案、提案和建议，主动接受人民群众及电子网络、新闻媒体等的社会监督，强化自我纠偏能力，不断改进政府工作。

　　弘扬务实之风。政府工作重在落实。要大力倡导不图虚名、不事张扬、多干少说、注重实效的良好作风，努力营造勇于创新、敢于负责、团结协作、共克难关的浓厚氛围。深入开展 “两转两提”活动，转变工作作风，改进文风会风，腾出更多时间和精力抓落实。把应对危机、支持发展的优惠政策落到实处，防止棚架。对群众关注的热点、难点问题，对事关新乡发展的重大问题，牢记在心、盯住不放，一件一件抓好落实，一步一个脚印地把事业推向前进。

　　加强廉政建设。反腐倡廉是人心所向，必须加强教育、健全制度、注重预防、惩治腐败。牢固树立过紧日子的思想，坚持勤俭办事，狠刹铺张浪费之风，规范公务接待行为，严格公费出国出境管理，年内公务购车、出国、接待经费要有明显下降，确保年度一般预算支出零增长，真正把有限的资金和资源用在保增长、促就业、改善民生、维护稳定的关键环节上。规范权力运行，深化公共资源交易体制改革，从源头上预防腐败。加强对重点领域、关键部位、特殊岗位的监督管理，坚决纠正损害群众利益的不正之风，坚决治理破坏发展环境的突出问题，坚决查处各类违纪违法案件，坚决严惩腐败分子。

　　各位代表！完成今后五年政府工作目标和今年各项任务，使命光荣，责任重大。让我们高举中国特色社会主义伟大旗帜，深入落实科学发展观，认真贯彻上级部署，在市委领导下，锐意进取，开拓创新，为跨越发展、富民兴市而努力奋斗！
